# The theranostic potentialities of bioavailable nanocurcumin in oral cancer management

**DOI:** 10.1186/s12906-022-03770-3

**Published:** 2022-11-24

**Authors:** Marwa M. Essawy, Mostafa M. Mohamed, Hanaa S. Raslan, Salma T. Rafik, Ashraf K. Awaad, Omneya R. Ramadan

**Affiliations:** 1 Department of Oral Pathology, Faculty of Dentistry, Alexandria University, Alexandria, 21521 Egypt; 2 Center of Excellence for Research in Regenerative Medicine and Applications (CERRMA), Faculty of Medicine, Alexandria University, Alexandria, 21521 Egypt; 3 Department of Clinical Pharmacology, Faculty of Medicine, Alexandria University, Alexandria, 21521 Egypt; 4 Department of Biochemistry, Medical Research Institute, Manager of Flow Cytometry and Medical Imaging Units at the Center of Excellence for Research in Regenerative Medicine (CERRMA), Faculty of Medicine, Alexandria University, Alexandria University, Alexandria, 21521 Egypt

**Keywords:** Apoptotic, Bioavailability, Curcumin, Luminescent, Nanocurcumin, Oral cancer, Squamous cell carcinoma

## Abstract

**Background:**

Oral cancer, one of the most common cancers, has unimproved 5-years survival rate in the last 30 years and the chemo/radiotherapy-associated morbidity. Therefore, intervention strategies that evade harmful side effects of the conventional treatment modalities are of need. Herbal therapy as a complementary preventive/therapeutic modality has gained attention. Curcumin is one of the herbal compounds possessing unique anticancer activity and luminescent optical properties. However, its low water solubility limits its efficacy. In contrast, curcumin at the nanoscale shows altered physical properties with enhancing bioavailability.

**Methods:**

The current study evaluated the impact of nanocurcumin as an anti-oral cancer herbal remedy, comparing its efficacy against the native curcumin complement and conventional chemotherapeutic. An optimized polymeric-stabilized nanocurcumin was synthesized using the solvent-antisolvent precipitation technique. After assuring the solubility and biocompatibility of nanocurcumin, we determined its cytotoxic dose in treating the squamous cell carcinoma cell line. We then evaluated the anti-tumorigenic activity of the nano-herb in inhibiting wound closure and the cytological alterations of the treated cancer cells. Furthermore, the cellular uptake of the nanocurcumin was assessed depending on its autofluorescence.

**Results:**

The hydrophilic optimized nanocurcumin has a potent cancerous cytotoxicity at a lower dose (60.8 µg/mL) than the native curcumin particles (212.4 µg/mL) that precipitated on high doses hindering their cellular uptake. Moreover, the nanocurcumin showed differential targeting of the cancer cells over the normal fibroblasts with a selectivity index of 4.5. With the confocal microscopy, the luminescent nanoparticles showed gradual nuclear and cytoplasmic uptake with apparent apoptotic cell death, over the fluorescent doxorubicin with its necrotic effect. Furthermore, the nanocurcumin superiorly inhibited the migration of cancer cells by -25%.

**Conclusions:**

The bioavailable nanocurcumin has better apoptotic cytotoxicity. Moreover, its superior luminescence promotes the theranostic potentialities of the nano-herb combating oral cancer.

**Supplementary Information:**

The online version contains supplementary material available at 10.1186/s12906-022-03770-3.

## Background

Oral cancer is a highly relevant problem of a global burden, representing the 10^th^ most common malignancy and the 15^th^ leading cause of death worldwide [1]. Approximately 90% of oral cancers are squamous cell carcinoma (SCC) [2]. Despite the advances in diagnostic techniques and the treatment modalities, the 5-year survival rate of patients with SCC did not improve in the last 30 years [3]. Moreover, the incidence and prevalence of SCC reveal increasing rates, particularly in younger persons under 45 [4].

Herbal medicine is one of the complementary and alternative medicine pillars, depending on the active ingredients of plants in the prevention and treatment of diseases. Worldwide, it is gaining widespread popularity, integrating herbal remedies into the mainstream healthcare systems [5]. In cancer prevention, natural products have attracted wide attention as they are cost-effective, readily available, non-toxic, and highly potent agents, with fewer adverse effects than synthetic drugs [6].

One of the promising herbal candidates is curcumin, the natural polyphenol widely used as an herbal supplement [7]. It was first isolated in 1815 from the rhizomes of Curcuma longa to be initially purified by Vogel Jr in 1842 and structurally explored by Melabedzka et al. in 1910 as diferuloylmethane (1,6-heptadiene-3,5-dione-1,7-bis (4-hydroxy-3-methoxyphenyl)-(1*E*,6*E*) [8]. Biomedically, curcumin has attracted attention in recent decades due to its therapeutic potential as an anti-inflammatory, anti-diabetic, and anti-aging agent. Furthermore, it has antioxidant, anti-mutagenic, and antimicrobial properties [9–11]. Moreover, curcumin exhibits a unique anticancer activity through the induction of apoptosis, inhibition of proliferation, and prevention of invasion, without delirious effects on the adjacent healthy cells [12–14].

Despite curcumin promising properties, it has poor water-solubility and low chemical stability, which are the major obstacles hindering its biological use. Curcumin -due to its hydrophobicity- tends to bind to the phospholipid of the cell membrane by hydrogen bond, resulting in low availability of curcumin inside the cytoplasm [15, 16]. After oral administration of 500 mg/Kg in an early in vivo study, curcumin has revealed a maximum plasma concentration of 0.06 ± 0.01 µg/mL [17]. In a clinical study using human volunteers, curcumin serum concentration one h post-administration of 2 g curcumin orally was either undetectable or very low (< 10 mg/mL) [18]. Our team is interested in using herbal medicine, including curcumin, in combating oral squamous cell carcinoma in vivo*.*However, curcumin has shown limited bioavailability, clinical efficacy, and low cellular uptake [19].

Curcumin, on the other hand, is soluble in different organic solvents. However, their biological uses are limited to the reported toxicity of these organic solvents. Therefore, curcumin water-solubility is still the target for biocompatibility and safety purposes. One of the most promising solutions for curcumin hydrophobicity is synthesizing curcumin at nanosize [10, 20]. In nanomedicine, improved pharmacokinetics and pharmacodynamics profiles of the nanosized drugs enhance their bioavailability [21]. Therefore, curcumin synthesis on the nanoscale would overcome its poor water-solubility problem, enhancing its bioavailability and therapeutic efficacy [22]. Moreover, the unique luminescent properties of nanocurcumin promote the safe utilization of this nano-herbal candidate in diagnostic nanomedicine [23]. Many researchers have demonstrated the anticancer activity of nanocurcumin. Ideally, the hydrophilic curcumin nano-formulation would enhance the anticancer efficacy of the native hydrophobic curcumin with a higher cancer cell selectivity than the adjacent normal cells [24].

Therefore, previously we optimized our nanocurcumin platform using the solvent antisolvent precipitation method [25]. We verified the parameters influencing the herbal nanoparticles' size, steric stability, biocompatibility, and luminescence. Herein, we tested the anti-tumorigenic efficacy of the nanocurcumin against oral squamous cell carcinoma cell line, investigating the impact of enhanced bioavailability of the hydrophilic nanoplatform. Besides the potency of nanocurcumin over its native complement, the nano-herbal candidate revealed further preferable apoptotic cell death with differential cancer-targeting over the nonspecific chemotherapeutic delirious necrotic reaction.

## Materials and methods

### Chemicals and cell lines

The purchased curcumin was from Alpha Chemika, India (Batch. No CM 487, Sr.No. AL 1575 00,005, HPLC 95%), while polyvinylpyrrolidone (PVP; Mw 40.000) and 3-(4,5-dimethythiazol-2-yl)-2,5-diphenyltetrazolium bromide (MTT) were from Sigma Aldrich, US. Acetone and all other reagents were of analytical grade and used as received. EIMC United Pharmaceuticals, Egypt commercially supplied doxorubicin (DOX).

We purchased the human oral cancer cell line SCC4 from the American Type Culture Collection (ATCC; Virginia, USA), while human gingival fibroblasts were isolated and donated by the Center of Excellence for Research in Regenerative Medicine and Application (CERRMA). Informed consent was obtained from all participants for isolation of gingival fibroblasts. Experiments were conducted following the guidelines approved by The Alexandria University Ethics Committee (IRBNO:00010556-IORG0008839).

Lonza (USA) was the source of Dulbecco's Modified Eagle's Medium (DMEM), penicillin, streptomycin, fetal bovine serum (FBS), trypsin/EDTA, and phosphate-buffered saline (PBS). Both cell lines' primary cultures were cultivated in DMEM (high glucose for SCC4, while low glucose for gingival fibroblasts) supplemented with 10% (v/v) FBS and 0.5% (v/v) antibiotics (100 U/mL penicillin and 100 U/mL streptomycin).

The dimethyl sulfoxide (DMSO) was available from Fisher Chemical (Fisher Scientific UK), whilst annexin V-fluorescein isothiocyanate (FITC)/propidium iodide (PI) for apoptotic assay was form BD Pharmingen, BD, Biosciences, USA. All reactive oxygen species (ROS) kits and antioxidant assays were supplied by Biodiagnostic, Egypt.

### Synthesis and characterization of curcumin nanoparticles

We followed our previously optimized method for nanocurcumin preparation based on the solvent-antisolvent precipitation method [25]. A 10 mg of curcumin powder dissolved in 1 mL acetone was added dropwise on stirring (500 rpm, at room temperature) to 15 ml deionized H_2_O, containing 0.5% w/v PVP as a stabilizer, till obtaining deep orange nanocurcumin suspension. Then the solution was lyophilized by Buchi, Lyovapor L-200 (Switzerland) for further characterization of the nano-powder.

The preliminary detection of nanocurcumin was through UV–Visible spectrophotometer (DeNovix, DS-11 FX + , US). The particle size, polydispersity index (PDI), and zeta potential of curcumin nanoparticles were assessed by the Zetasizer Nano ZS (Malvern Instruments, Worcestershire, UK), with a dilution ratio of 1:6.

Morphology and size of nanocurcumin were analyzed by transmission electron microscope (TEM; JOEL, JSM-6360LA, Japan). Meanwhile, the assessment of nanocucrcumin luminescence was performed at 240 nm exciting wavelength using fluorescence spectrophotometry (Agilent Technologies, Malaysia) [26].

Surface chemistry analyses of curcumin, PVP, and nanocurcumin were performed using a Fourier transforms infrared (FTIR) spectrometer (PerkinElmer Inc, Shelton, CT, US), with a screening spectrum of 4000–500 cm^−1^.^25^ Further characterizations of the synthesized nanocurcumin concerning solubility test and standard curve are provided in Supplementary materials.

### IC_50_ and cytotoxicity

The cytotoxic effect of nanocurcumin versus curcumin on SCC4 cells was evaluated by the MTT assay, taking human gingival fibroblasts as a positive control cell line to determine cell specificity [27]. The cells from both lines seeded onto a 96-well plate with a density of 7000 cells/well were treated with 25 – 250 µg /mL serial concentration of DMEM-resuspended nanocurcumin and acetone-dissolved curcumin. DOX-treated cancer cells were positive control. After 24 h incubation, 100 µL/well MTT (0.5 mg/mL DMEM) was added and incubated at 37 °C for 3.5 h. After MTT removal, the formazan crystals produced by viable cells were dissolved by 100 µL/well DMSO. We used a microplate scanning ELISA reader (Infinite F50, TECAN, Switzerland) to measure cell viability by quantifying the optical density of the DMSO at 570 nm. The percentage of cell viability calculations follows: $Viable \ cell \%=\left({OD}_{treated}/{OD}_{untreated}\right)\times 100$

Cell specificity of SCC4 versus gingival fibroblasts for the proposed treatments depends on the following [28]: $Cell \ specificity \ = \ {Gingival \ fibroblasts \ IC}_{50}/{SCC4 \ IC}_{50}$.

### Apoptosis assay

Apoptosis detection was done through a flow cytometer using FITC-Annexin V/PI co-staining technique to differentiate between living, apoptotic, and necrotic cells. With a seeding density of 30 × 10^4^ cells / well onto a 6-well plate, SCC4 cells were treated with the IC_50_ of the proposed treatments (nanocurcumin, curcumin, and DOX) for 4 and 24 h. After trypsinization, the cells were stained with 10 µL Annexin V-FITC and incubated for 30 min at 4 °C. The cells were further incubated with 5 µL PI at dark and immediately analyzed by a flow cytometer (BD, Bioscience, US). The data evaluation was through FACS Diva software (BD Bioscience, US) [29].

### Cytological evaluation

We performed a smear from the treated SCC4 cells to assess the individual nuclear and cytological morphological alteration after nanocurcumin treatment. Trypsinized treated cells grown on a 6-well plate were fixed with ethanol and smeared on glass slides for H&E staining procedures. The cytological examination was performed at × 200 and × 400 powers using Olympus DP20 digital camera joined to an Olympus BX41 microscope [30].

### Scratch wound healing assay

For assessing the anti-migratory efficacy of curcumin nanoparticles on SCC4, cells were grown in 6-well culture plates at a seeding density of 25 × 10^4^ / well to achieve the confluent monolayer. Then, the cell monolayer was gently scratched with a sterile 200-μL pipette tip to make one straight cell-free line. After PBS wash, cells were treated with nanocurcumin and curcumin IC_50_, taking scratched wells treated with DOX as a positive control.

Wound healing was monitored and measured at 0, 24, and 48 h. The images (5 images /well) of the wound were captured at a magnification of × 100 using an inverted microscope (Olympus, Japan). The horizontal distance of the wound gap was measured using Image J (version 1.53C, NIH, US) to determine the cell migration rate and percentage of wound closure according to the following equation:  $\% Wound \ closure=100-[\left({A}_{t}/{A}_{0}\right)\times 100]$, where *A*_*t*_ is the wound area at time t and *A*_*0 *_is its initial area [31].

### Oxidative stress determination

Following 24 h of SCC4 treatment with nanocurcumin, we assessed both arms of oxidative stress: ROS and antioxidant productions. Malondialdehyde (MDA; nmol/g), nitric oxide (NO; µmol/L), and hydrogen peroxide (H_2_O_2_; mM/g) were measured, along with the total antioxidant capacity (TAC; mM/L) and reduced glutathione (GSH; mg/g). Normalization of each measurement was in comparison to the control readings. ROS determination using colorimetric assays was according to the manufacturer's instructions [29].

### Cellular visualization of nanocurcumin

For the cellular homing track of curcumin nanoparticles, SCC4 cells plated on glass coverslips and incubated with treatments for 24 h were examined by confocal microscopy, taking untreated plates as control. The cells were washed with PBS and fixed using 4% paraformaldehyde. Then, the cells were permeabilized with Triton X (0.2%), followed by counterstaining using Hoechst for 10 min. We examined the glass slides using confocal laser scanning microscopy (Leica TCS SPE, Germany) equipped with imaging software (Leica LASX, Germany). The fluorescence intensity was analyzed morphometrically using an image analysis software (Image J; 1.52p software 32, NIH, USA) [25, 32].

Analysis of the fluorescence intensity of ZnS NPs was following the equation:

$$Corrected \ total \ cell \ fluorescence=Integrated \ density-\left(Area \ of \ selected \ cell \times Mean \ fluorescence \ of \ background\right)$$

### Statistical analysis

Expression of experimental and in vitro results are as mean ± SD of 3 independent experiments, with triplicates for each investigation. With a level of significance determined at *p* < 0.05, we performed the statistical analyses using GraphPad Prism version 8.0.1 (GraphPad Software, San Diego, California, USA).

Regression analysis was the test used to determine the half-maximal inhibitory dose of the proposed treatments. The two-way ANOVA followed by Tukey’s multiple comparisons were the tests of significance used for flow cytometry, scratch wound healing, and fluorescent intensity analyses. Meanwhile, the significant test of the oxidative stress was one-way ANOVA.

## Results

### Synthesis of highly stable auto-luminescent hydrophilic curcumin nanoparticles of homogenous size distribution

**Fig. 1:**
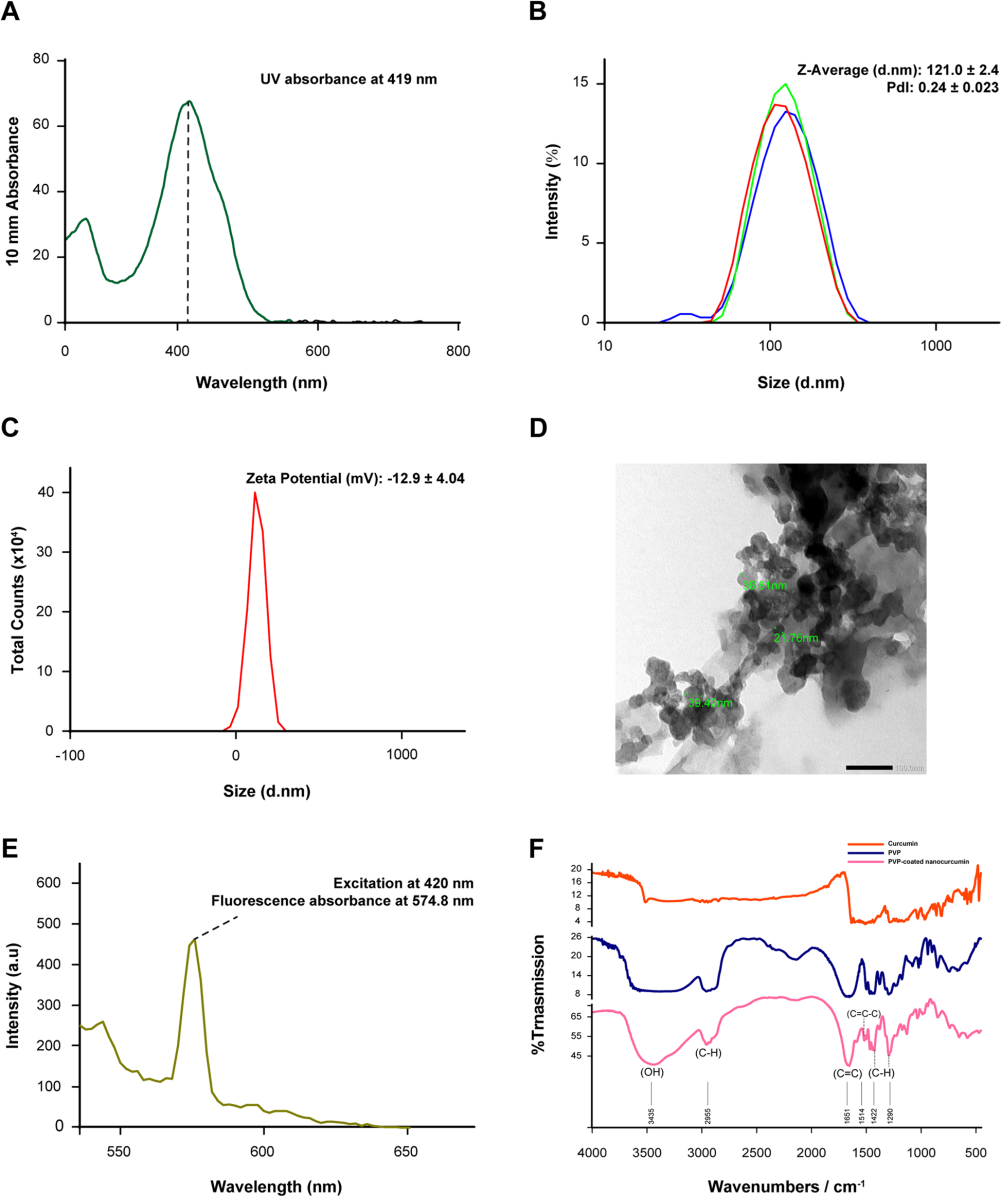
The different characterization assays of PVP-stabilized curcumin nanoparticles. **A** UV–vis spectrophotometer with a smooth regular specific absorbance peak at 419 nm. **B** and **C** The dynamic light scattering results ensure the monomodal uniformly distributed PVP-stabilized nanosuspension. **D** Shows the TEM results, revealing spherical mono-dispersed particles. **E** The robust luminescent curcumin nanoparticles record a sharp fluorescent peak detected at 574 nm. **F** The FTIR confirms the success of the coating process by comparing PVP-stabilized nanocurcumin against pure PVP

The addition of curcumin dissolved in acetone dropwise to the deionized H_2_O on a stirring for one minute at 500 rpm precipitated the nanocurcumin as a clear yellow nanosuspension without precipitate. The UV–Vis spectrophotometer results of PVP-stabilized nanocurcumin revealed a narrow, smooth, and regular peak with high yielding capacity at the characteristic absorbance peak of nanocurcumin at 419 nm (Fig. 1A).

Coating curcumin nanoparticles with 0.5% PVP resulted in a homogenous mono-dispersed nanoparticles population with an average size of 121 ± 0.03 nm and a low PDI of 0.2 ± 0.02. Furthermore, the surface charge of -12.9 mV indicated the stabilization of our nanosample (Fig. 1B, C). The TEM analysis of PVP-stabilized nanoparticles showed widely distributed rounded particles with a mean size of 33.33 ± 10.1 nm (Fig. 1D). Meanwhile, excitation of nanocurcumin at 420 nm unraveled their potent autofluorescence, with a sharp absorbance peak at 574 nm (Fig. 1E). Furthermore, bringing the curcumin from its rude native bulk form to the nanosize changed the physical properties of the hydrophobic nature of the former to a readily soluble form of the latter. In testing the solubility of the synthesized nanocurcumin, the PVP-stabilized nanoparticles were highly soluble in water and DMEM without any precipitate. In contrast, the solubility test proved the poor solubility of curcumin powder in water as well as in DMEM (Supp. Fig. 1A).

According to the analytical curve in Supplementary Fig. 1B, the PVP-stabilized nanosuspension revealed the superior yielding capacity of ~ 36.7 mM, compared to the initial 2.7 mM of curcumin powder.

The FTIR results confirmed the success of synthesizing biocompatible PVP-coated curcumin nanoparticles. Comparing the simple shift in the absorbance spectrum between curcumin powder and nanoparticles in Fig. 1F indicates minor structural changes during the solvent anti-solvent technique. Moreover, the absence of ketone peaks for acetone in the range of 1725–1700 cm^−1^ confirms the biocompatibility of the nanocurcumin. On the other hand, the prominent vinyl C-H stretches at 1422 and 1290 cm^−1^ in the nanocurcumin, compared to the corresponding ones in pure PVP, indicated the success in stabilizing the nanocurcumin.

### The potency of nanocurcumin anti-tumorgenicity and cancerous specificity

**Fig. 2:**
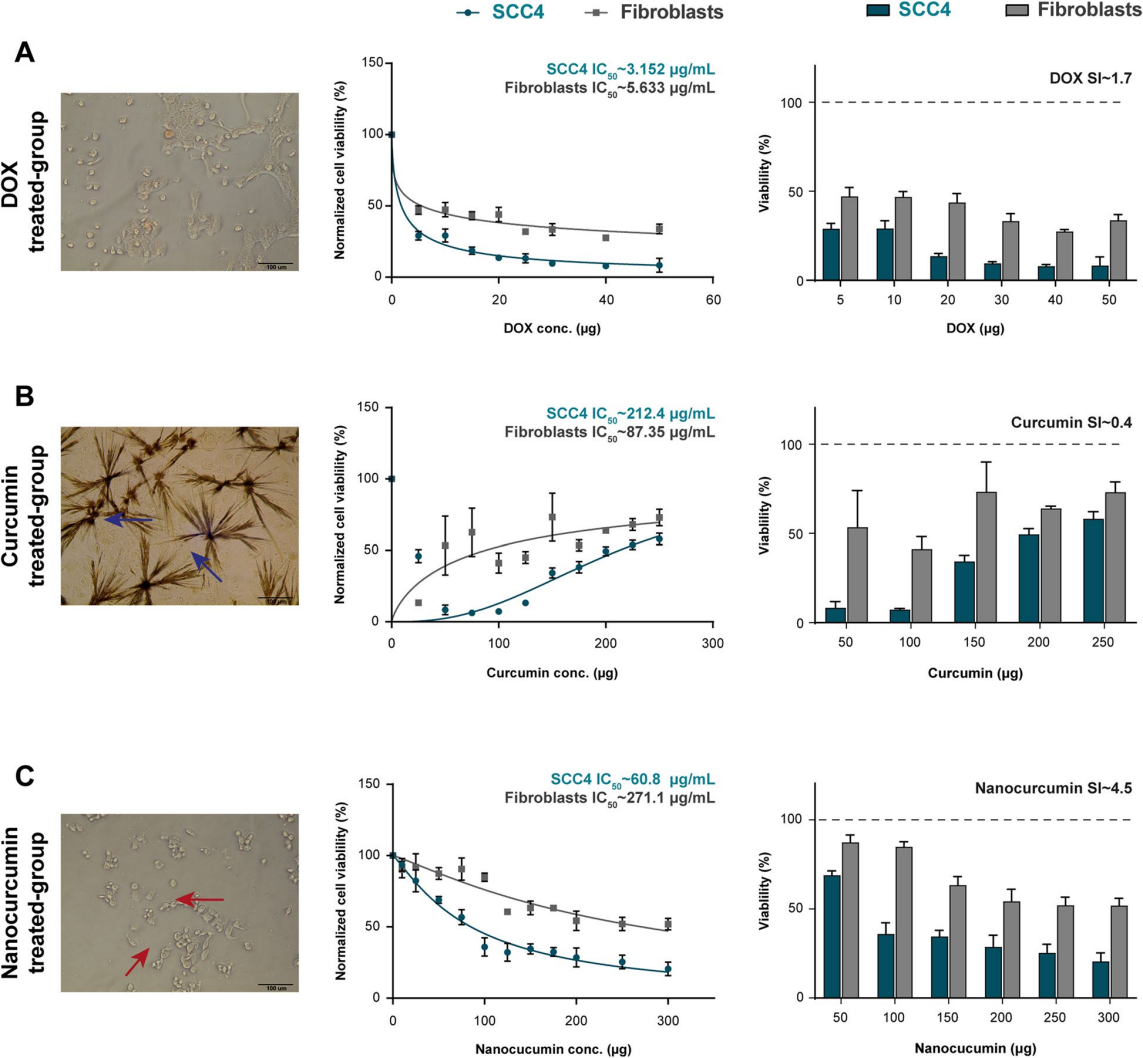
The cytotoxic effects of the proposed treatments on cancer SCC4 and normal gingival cells, where nanocurcumin shows tumor cell specificity by 4.5. **A** The phase-contrast morphology of DOX-treated SCC4 shows small rounded detached cells with pyknotic nuclei and thin perinuclear cytoplasmic rims. The dose-dependent curve and the bar chart reveal the sharp drop of IC_50_ to ~ 3.1 µg/mL for SCC4 versus ~ 5.6 µg/mL for gingival cells. **B** The inverted light microscope photomicrograph of the curcumin-treated SCC4 demonstrates the distinctive crystalline form of curcumin (blue arrows), which hinders its cytotoxic effects, a reflected picture in the cell viability dose curve and plot chart. Upon increasing curcumin concentration, the SCC4 cells regain their viability due to the formation of the crystals with IC_50_ of ~ 212.4 µg/mL. **C** Nanocurcumin-treated SCC4 cells show the blebs (red arrows) formed on the cell surface, denoting the generation of apoptotic bodies. The bar chart reveals the gradual decrease in SCC4 cell viability, where the IC_50_ curve confirms the dose-dependent cytotoxic effect of nanocurcumin, calculating ~ 60.8 µg/mL. Data presentation is as mean ± SD of three independent experiments. The digital microscopic photomicrographs are captured using Olympus CKX41 phase-contrast microscope at × 200 of a scale bar 100 µm

Compared with the untreated prickle squamoid epithelial cancer cells (Supplementary Fig. 2), the DOX-treated SCC4 cells showed dramatic morphological changes after 24 h of treatment. The cells detached from each other and became small, rounded with shrunken nuclei surrounded by a thin perinuclear cytoplasmic rim. The potent DOX cytotoxic effect began at the lowest concentration of 5 µg/mL, revealing a decrease in cell viability to 29.1 ± 3.0%. Increasing the DOX concentration to 50 µg/mL further decreased the cell viability remarkably to 8.3 ± 4.8%. The IC_50_ of DOX for SCC4 after 24 h-incubation was ~ 3.15 µg/mL (Fig. 2A).

The SCC4 treated with acetone-dissolved curcumin did not show the morphological changes observed in the DOX-treated group. Instead, the cells became swollen with shrunken nuclei, abundant cytoplasm, and the characteristic perinuclear halo. Interestingly, curcumin made a crystalline form with acetone upon increasing its concentration. These crystals inhibited curcumin uptake by the cell, hindering its cytotoxic effects. These findings were consistent with the pattern of cell viability, which was not in a dose-dependent manner. The completely dissolved curcumin up to 150 µg/mL revealed a notable decrease in cell viability to 34.3 ± 3.4%, a reversed pattern retrieved upon increasing curcumin concentration. At 200–250 µg/mL, the cell viability returned higher to 59.4 ± 3.1% and 58.1 ± 4.07%, respectively, with a calculated IC_50_ of ~ 212.4 µg/mL (Fig. 2B).

The 24 h morphological changes of nanocurcumin-treated SCC4 cells included cell detachment and nuclear size reduction. Furthermore, cell shrinkage and the formation of blebs on the cell surface resulted in the generation of apoptotic bodies. The cytotoxic effect of nanocurcumin was in a dose-depended manner. Initial cell sensitization started at the lowest nanocurcumin concentration of 25 µg/mL, which revealed inhibitory effects on cell viability to 81.9 ± 1.5%. By increasing the nanocurcumin to 150 and 200 µg/mL, the cell viability decreased gradually to 34.5 ± 3.5% and 28.6 ± 6.6%, respectively, with IC_50_ of ~ 60.8 µg/mL (Fig. 2C).

Comparing the IC_50_ amongst SCC4 and gingival fibroblasts, the normal cells were more resistant to DOX and nanocurcumin, recording 5.6 µg/mL and 271.1 µg/mL, respectively. However, the chemotherapeutic did not show differential selectivity between cancerous and normal cells as potent as the nano-herbal medication, where nanocurcumin revealed a ~ three fold increase in its selectivity index than DOX. Meanwhile, fibroblasts were more vulnerable to the acetone solvent of the curcumin with lower IC_50_ than that of SCC4, recording ~ 87.35 µg/mL (Fig. 2).

### The favorable apoptotic effect of nano-herbal medicine versus the necrotic outcome of the chemotherapeutic

**Fig. 3:**
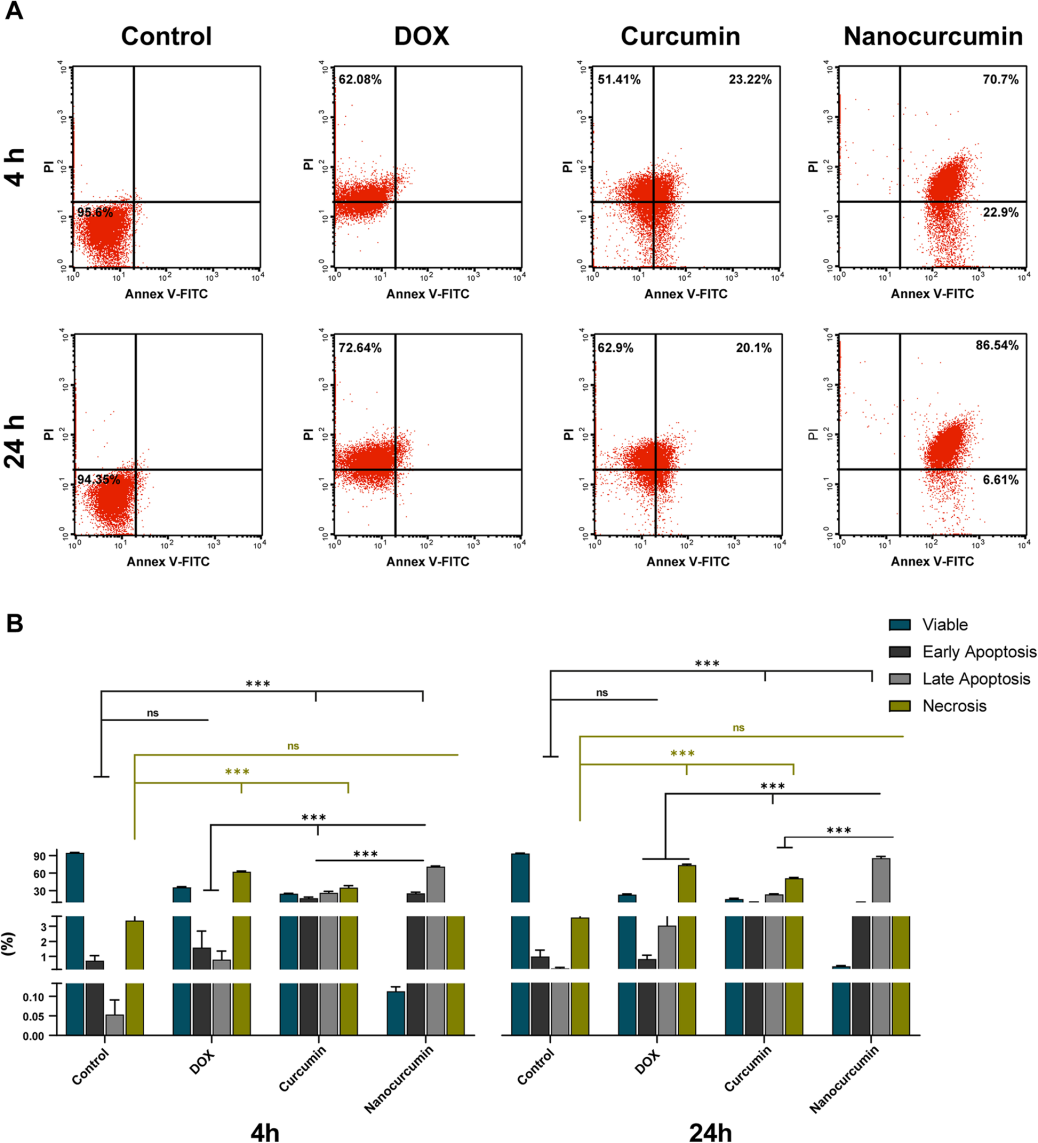
Detection of different cell deaths after treating SCC4 by flow cytometer assay after four h and 24 h incubation periods. **A** The DOX-treated group reveals the necrotic effect at both intervals. Meanwhile, curcumin presents diverse effects on SCC4 cells, where the early stage reveals late apoptosis and necrosis, while the late stage shows elevated necrotic cell deaths. On the other hand, nanocurcumin exerts a prominent apoptotic effect on SCC4 cells at both incubation periods. **B** Blot charts demonstrate the flow cytometer results at early (4 h) and late (24 h) incubation periods. Cancer cells treated with DOX and curcumin reveal a drop in SCC4 viability caused by the significant necrosis at both intervals. Meanwhile, the decreases in cell viability by nanocurcumin are due to the profound increases of early and late apoptotic indices at both timelines. The data are the mean ± SD of three independent experiments. Asterisk denotes statistical significance, where *** (*p* < 0.001). While ns implies not significant with *p* > 0.05

The flow cytometer assay revealed variations in apoptosis detection between groups, with the results summarized in Fig. 3.

The SCC4 cells treated with 3.1 µg/mL DOX revealed profound necrotic cell death. After four h of DOX treatment, the cell viability dramatically decreased to 35.3 ± 1.3% (*p* < 0.001) with a shooting increase in the necrosis to 62.1 ± 1.3% (*p* < 0.001), in comparison with the untreated SCC4 cells. DOX after 24 h incubation with SCC4 further reduced cell viability to 22.8 ± 1.3% with the concomitant significant increase of necrotic population to 73.5 ± 1.5% (*p* < 0.001).

The SCC4 cell death induced by IC_50_ of acetone-dissolved curcumin varied between necrosis of 54.3 ± 2.7% and late apoptosis of 21.3 ± 2.03% after four h incubation. This pattern changed after 24 h of curcumin treatment, where most of the cells became necrotic (61.4 ± 1.5%) with similar apoptotic detected population.

Treatment with 60.8 µg/mL nanocurcumin revealed a significant reduction of the viable cells at both four h and 24 h intervals to 0.11 ± 0.01 and 0.3 ± 0.05% (*p* < 0.001), respectively. The resultant cell death was translated mainly as apoptosis, tangling between early and late apoptosis, with almost undetectable necrosis. After four h, nanocurcumin recorded a significant increase in the early and late apoptosis at 25.2 ± 2.1 and 70.9 ± 1.1% (*p* < 0.001), respectively. With increasing the incubation time to 24 h, most of the cell population was shifted to the late apoptosis, recording 85.5 ± 2.8%.

**Fig. 4:**
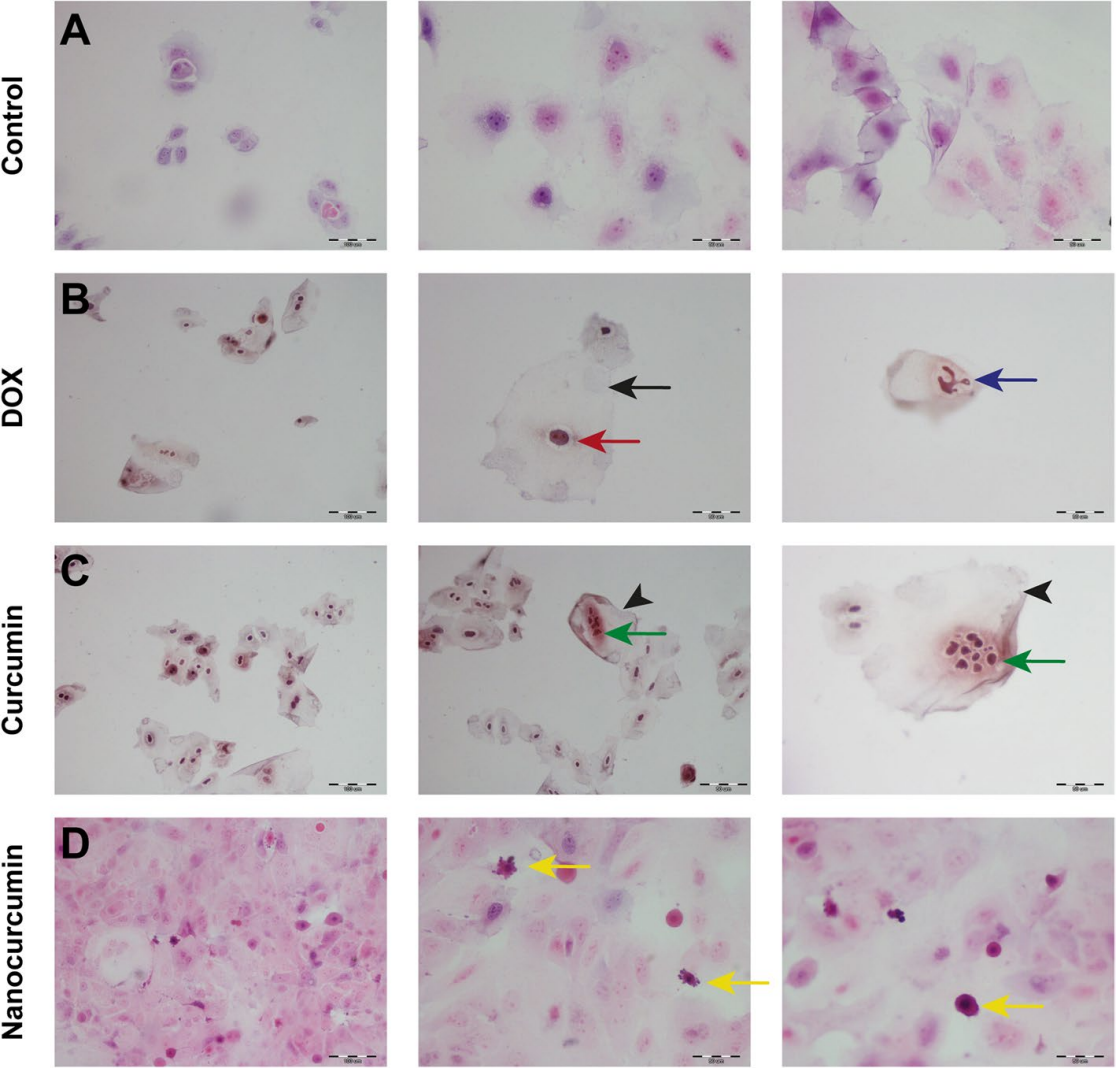
A light microscope photomicrograph of H&E-stained cytological smears from different treated SCC4 groups. After 24 h incubation with DOX, the squamous prickle-shaped cells in (**A**) become swollen, demonstrating ballooning degeneration (black arrow) with disproportionated shrunken (red one) karyolitic nuclei (blue arrow) in (**B**). **C** The equivalent ghost-like (black arrowheads) cytological deteriorations retrieved from curcumin treatment confirms the necrotic cell death effect exerted by the acetone. Green arrows point out the distinctive nuclear karyorrhexis. **D** Meanwhile, the enhanced solubility of nanocurcumin induces apoptotic cell death, resulting in small-sized, deeply stained apoptotic bodies (yellow arrows) with pyknotic nuclei and surface blebs. Scale bar 100 µm equals × 200 magnification, while 50 µm is equivalent to × 400

In conformation to the previous results, cytological examination revealed the predominant necrotic effect of DOX versus the apoptosis induced by curcumin nanoparticles. Figure 4 displays the alteration array of the prickle-shaped untreated SCC4 cells to ballooning degeneration upon DOX treatment with karyolysis. A similar retrieved picture upon curcumin treatment, where the acetone solvent ruptured the cell membrane, inducing abrupt nuclear fragmentation. Meanwhile, bringing curcumin to the nanosize with enhanced bioavailability results in apoptotic cell death. The cancer cells reveal deeply eosinophilic shrunken cytoplasm, pyknotic nuclei with condensed chromatin, and preserved nuclear membrane. These apoptotic bodies show multiple surface blebs.

### The sturdy anti-migratory efficacy of the nanocurcumin with potent antioxidant ability

**Fig. 5:**
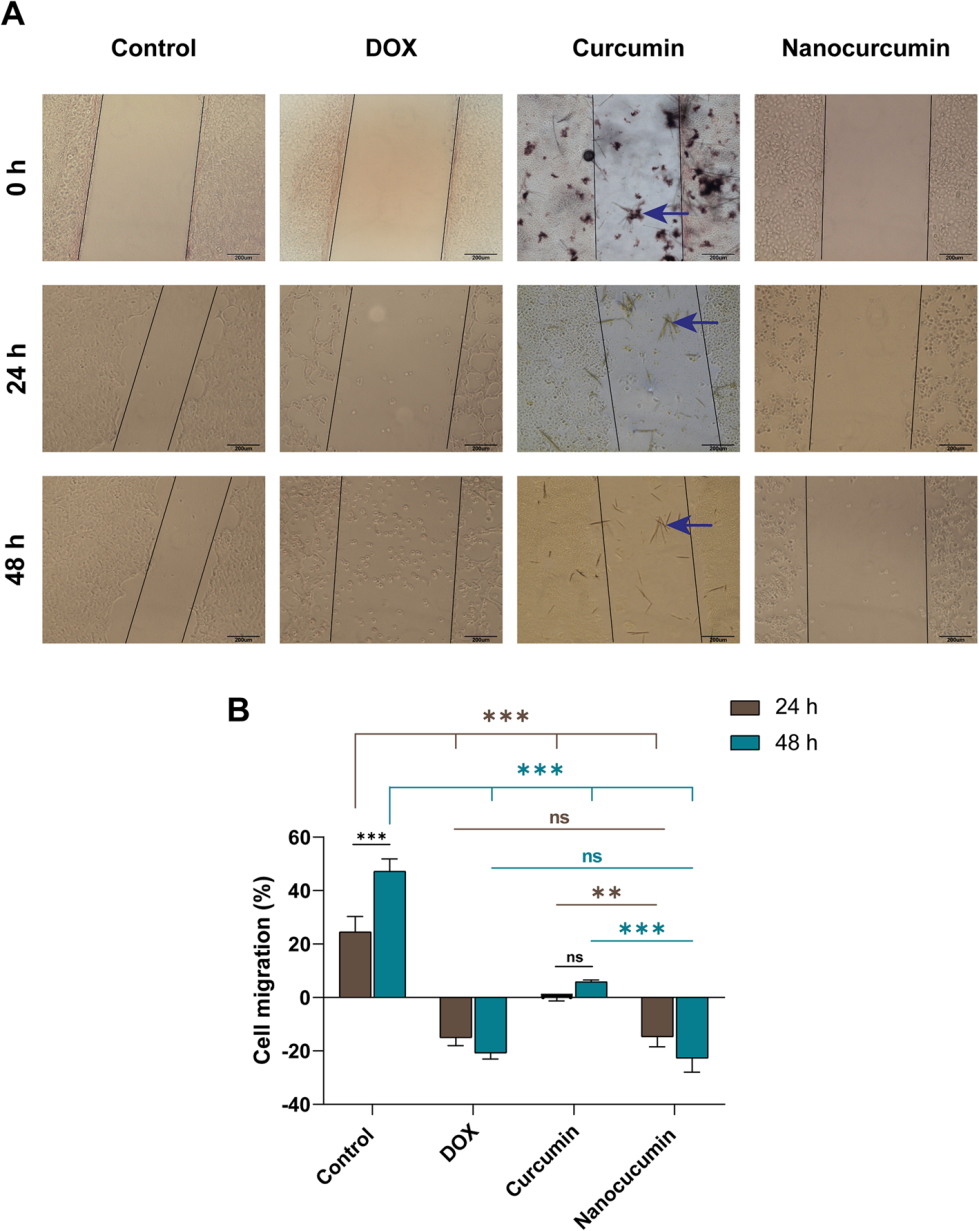
The anti-proliferative capability of the different treatment modalities against SCC4. **A** A contrast phase inverted microscope photomicrograph shows the scratch wound healing assay of SCC4 after 24 and 48 h treatment, taking 0 h as base time. At both intervals, nanocurcumin inhibits cell migration and further increases the scratch line patency as efficiently as DOX. Meanwhile, the insoluble crystals (blue arrows) hinder the anti-migratory effect of curcumin in its bulk form. The plot chart in (**B**) reveals the percentage of cell migration inhibition in treated groups compared with the proliferative behavior of the untreated cells. The data are the mean ± SD of three independent experiments. Asterisk denotes statistical significance, where ** (*p* < 0.01) and *** (*p* < 0.001). While ns implies not significant with *p* > 0.05

The scratch wound healing assay unraveled the anti-proliferative potentiality of the different treatment modalities. At 0 h, the scratch line of each group showed an insignificant difference (*p* > 0.5) in the gap, indicating the consistency of the scratch technique (Fig. 5).

After 24 h, the proliferative tendency of the untreated cancer cells was evident by the significant closure of the cell-free zone by 24.5 ± 5.7%. In the DOX group, the chemotherapeutic exerted its anti-tumorigenic effect by inhibiting SCC4 migration and increasing the width of the cell-free zone by -15.1 ± 2.9%. Meanwhile, the curcumin-treated SCC4 cells did not show a comparable anti-proliferative effect as DOX, attributed to the crystalline structures formed by acetone. In contrast, the bioavailable solubilized nanoparticles inhibited cell proliferation, preserving the cell-free zone. The patency of the scratch line in the nanocurcumin group was similar to that of the DOX group (*p* > 0.05, Fig. 5).

The time-dependent effect was conspicuous after 48 h of treatment, where the untreated cells continued to migrate, closing the cell-free zone significantly by 47.2 ± 4.6% (*p* < 0.001). The insoluble curcumin crystals hindered the bioavailability of curcumin, freezing the wound zone as it was before 24 h (*p* > 0.05). The picture was completely different in DOX and nanocurcumin groups with patent scratch lines. The comparable cytotoxic effect of DOX and nanocurcumin led to cell death with almost any remaining cells (Fig. 5).

**Fig. 6:**
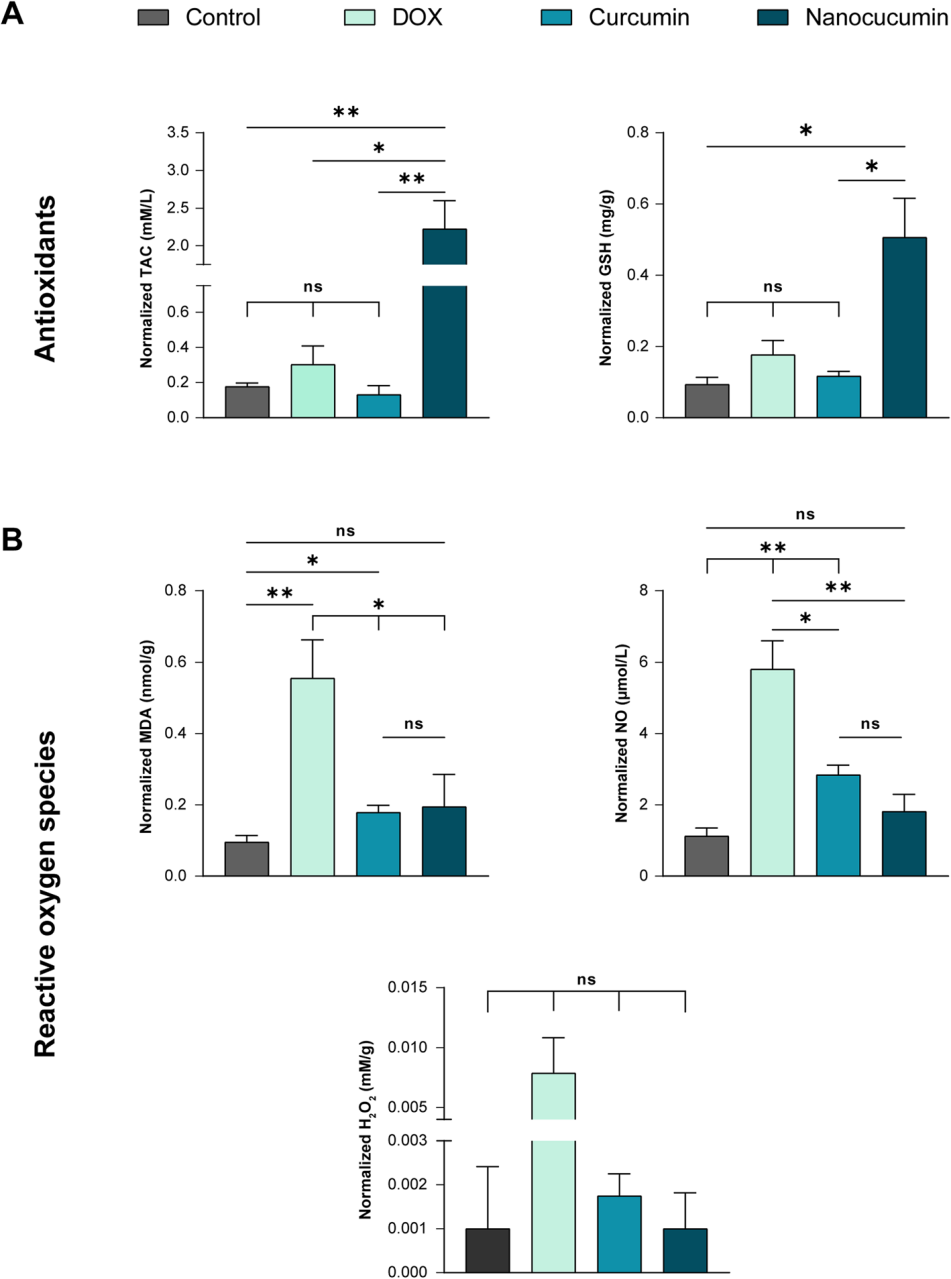
Bar charts of the oxidative stress arms, antioxidant capacity (**A**) versus ROS production (**B**), after 24 h SCC4 treatment. DOX stressed cancer cells by powerful ROS release, in contrast to the antioxidant nano-herbal remedy scavenging ROS. The curcumin stands a step behind chemotherapeutic in stressing the SCC4 oxidatively. The data are the mean ± SD of three independent experiments. Asterisk implies statistical significance, where * (*p* < 0.05) and ** (*p* < 0.01). While ns denotes not significant with *p* > 0.05

Evaluating the oxidative stress produced by the proposed treatments, there was a counter difference between ROS production and clearance in each modality (Fig. 6). The ability of nanocurcumin to act as a ROS scavenger stands behind its cytotoxicity, where the nano-herbal therapy produced a significant amount of normalized antioxidants (TAC and GSH) in comparison with untreated SCC4 cells (*p* < 0.05). Meanwhile, chemotherapeutic induced oxidative stress with ROS production took the upper hand over their removal ability. In contrast to the nanoplatform, DOX released considerable values of ROS candidates, recording normalized 0.55 ± 0.1 nmol/g MDA and 5.8 ± 0.78 µmol/L normalized NO. The curcumin was similar to the DOX in oxidative stress induction but to a lesser extent, where the acetone-dissolved curcumin was midway tangling between modest MDA producer (*p* < 0.05) to potent NO releaser (*p* < 0.01) compared with untreated SCC4.

### In situ tracking of the theranostic nano-herbal candidate thanks to their luminescence properties

On confocal laser scanning microscopy examination, the autofluorescence of nanocurcumin confirmed the fluor-spectrophotometer results (Fig. 1E). Compared with the red fluorescent DOX, the green luminescence of curcumin enabled its cellular uptake tracing. Moreover, the fluorescence properties of proposed treatments boosted the evaluation of the cytological and nuclear alterations.

The SCC4 showed intense nuclear homing of DOX after four h and 24 h of its application, with fluorescence indifference between the two examination periods (*p* > 0.05). The untreated SCC4 changed their nuclear pattern with the nuclear specificity of the chemotherapeutic. Whether early or late, targeting the nucleus with DOX resulted in pyknosis and condensed heterochromatin.

The curcumin showed altered confocal results, especially in its insoluble bulk form. Rapidly after four h, the acetone dissolved the cell membrane phospholipid, resulting in a matted cytological appearance of the cancer cells with in-differential localized curcumin particles. The intense gush of fluorescent curcumin in both intervals was traced as cytoplasmic and nuclear, with the latter showing complete lysis.

**Fig. 7:**
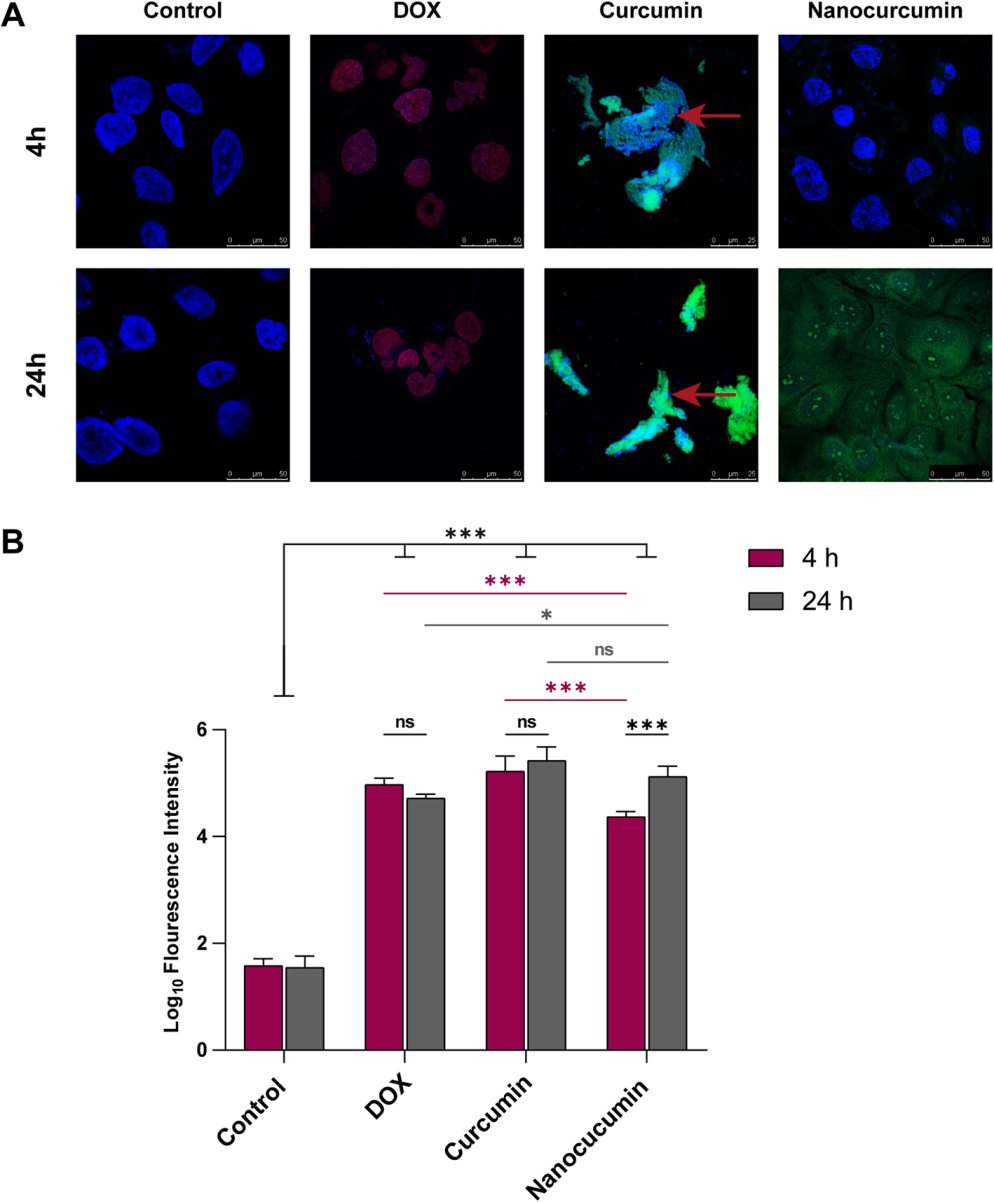
Evaluation of the curcumin fluorescent intensity in its bulk and nano forms with cellular alterations determination, taking the DOX luminescence as a positive control. **A** The confocal scanning microscopy images reveal the nuclear and cytoplasmic localization of the nanocurcumin versus the nonspecific uptake of the curcumin macroparticles, whereas the DOX shows nuclear differential uptake. **B** The histomorphometric analyses of the fluorescence intensities, where the luminescent nanocurcumin shows a gradual increase*** in its uptake between 4 and 24 h. Meanwhile, DOX and curcumin reveal intense fluorescent signals regardless of the incubation time with drastic cytological deterioration of the necrotic matted SCC4 (red arrows in **A**). The data are the mean ± SD of triplicate per each time interval. Asterisk of * denotes *p* < 0.05 and ** implies *p* < 0.05, while ns of *p* > 0.05 points out not significance

On the other hand, the bioavailability of curcumin at the nanoscale enabled their gradual uptake. After four h, the luminescent nanoparticles were spotted in the cytoplasm. While after 24 h, the nuclear homing together with cytoplasmic localization was significantly evident in the auto-fluorescent nanoparticles (*p* < 0.001), which enabled visualization of their cytotoxic effects. The enhanced solubility of the nanocurcumin promoted its uptake by SCC4 cells by preserving their cytosolic and nuclear membranes, indicating the apoptotic effect of the nano-herbal candidate. Confirming this favorable cytotoxic effect, the nuclei of the cancer cells showed chromatin fragmentation within their intact nuclear envelope (Fig. 7 and Supp Fig. 3).

## Discussion

Oral squamous cell carcinoma remains one of the most challenging malignancies to treat because of its high ability for direct invasion and metastasis. Despite the advances in therapeutic strategies, the prognosis is still poor. Furthermore, the morbidity associated with the radio/chemotherapy off-target is an added disadvantage [33]. Complementary medicine provides an alternative stream, depending on the active ingredients of herbs in the prevention and treatment of diseases [5]. In cancer management, curcumin, a natural compound, has proven its anti-tumorigenic activity. However, curcumin has a decreased biomedical efficiency due to its poor water-solubility [7].

Nanotechnology is an innovative science used to overcome the problems of reduced stability and bioavailability associated with poorly soluble drugs [20]. Therefore, vast techniques have been attempted to synthesis curcumin at nanoscale to maximize its biomedical beneficiary effect ranging from encapsulation of curcumin into different types of nanoparticles resulting in nanosuspension [34–36], to precipitation of the nanocurcumin by ultrasonication synthesizing nanopercipitate [37, 38]. However, these techniques are in need of complex operating circumstances and of expensive production costs because of high pressure and extreme low temperature requirements. Moreover, the time needed for curcumin release from the encapsulated nanoplatforms as well as the relative water solubility of the ultrasonicated nanoprecipitate encourage the search of more simple method of readily soluble nanocurcumin synthesis [39].

The solvent-antisolvent precipitation is another relatively simple, cost effective, and easy to scale-up method [39]. We, therefore, optimized our nanocurcumin preparation using the solvent-antisolvent precipitation method to credit our nanoplatform as a nano-herbal candidate combating SCC in vitro. First, we verified the variables included in this technique that directly influence the particle size, stability, and dispersity of the resulted nanopopulation. The UV–Vis spectrophotometer confirmed the synthesis of curcumin nanoparticles with a narrow, smooth regular peak of a specific absorbance at 419 nm, which is the characteristic peak of nanocurcumin [40].

Upon stabilizing the nanocurcumin with 0.5% PVP, the particle size reached 121 ± 0.03 nm with homogeneously distributed nanoparticles. The PVP migrates immediately to the hydrophobic surface of the newly formed curcumin nanoparticles, preventing their growth with resultant small particle size. Moreover, increasing the molecular weight of the stabilizer would increase its flexibility and elasticity, which in turn improves its ability to surround and make a full inclusion of the newly synthesized curcumin nanoparticles inside it [41].

The PVP in the current study formed an acceptable negative surface charge (-12.9 mV) around curcumin nanoparticles, providing steric stabilization that prevented nanoparticle growth and aggregation. Gelatin, another stabilizer creating a high negative surface charge (-30 mV) around the newly synthesized curcumin nanoparticles, has arrested their up-building but did not prevent their aggregation [42]. The proven water-solubility of the synthesized PVP-stabilized nanocurcumin would enhance its bioavailability and cellular uptake, improving its clinical efficacy. Moreover, the surface chemistry analysis ensured the biocompatibility of our nanoplatform by assuring the evaporation of the chosen acetone. From the screening of curcumin solubility in various organic solvents, acetone was the best verified one. Biologically, acetone is safe and can be easily removed from the final formulation by evaporation due to its low boiling point of 56 °C [42].

Our PVP-stabilized herbal nanocandidate was then authenticated for further anticancer investigations against oral SCC4 versus curcumin in its bulk form. DOX, an anthracycline antibiotic, was the positive control due to its comparable water solubility and auto-luminescent properties to nanocurcumin. The cytotoxicity results of the present study revealed the dose-dependent manner of the nanocurcumin IC_50_, 60.8 µg/mL. Besides, microscopic examination of the nanocurcumin-treated revealed rounded detached cells, a similar picture to DOX. The comparable cytotoxic effect of nanocurcumin to chemotherapeutic has been previously reported against esophageal and breast cancer cells using paclitaxel and 5-fluorouracil, respectively [43, 44].

Despite the IC_50_of nanocurcumin being higher than DOX (10.5 µg/mL), the documented cumulative cardiotoxicity of DOX and the development of drug resistance are the major obstacles facing its therapeutic efficiency. Moreover, the acute inhibitory efficiency of doxorubicin impaired its clinical implementation due to its unavoidable delirious reactions against surrounding normal cells [45]. Therefore, weighing the benefits of using herbal natural compounds tips the cuff towards the nanocurcumin against the cardio-toxic DOX.

In contrast to the curcumin precipitated crystals, the enhanced water/DMEM solubility of nanocurcumin facilities its uptake to exert its cytotoxic effect at a lower concentration, increasing its efficacy and bioavailability with decreasing its IC_50_. Furthermore, the biocompatibility effect of water-soluble nanocurcumin overcomes the cytotoxic effect gained from dissolving curcumin in organic solvents. The results of previous studies have confirmed the prompt cytotoxicity of nanocurcumin against different cancer cells with reduced IC_50_ values [46–48]. Moreover, combining nanocurcumin with berberine has receded the IC_50_ of the latter, displaying a synergistic anticancer efficacy in fighting breast cancer cells [49].

Similarly, nanocurcumin has revealed its dose- and time-dependent cytotoxic effects against hepatocellular carcinoma cell line. It has augmented transforming growth factor-β1 72 h post-treatment, reducing telomerase expression [46]. In esophageal adenocarcinoma cell lines, nanocurcumin, besides inhibiting cancer cell proliferation in a dose- and time-dependent manner, has potentiated the immune response to the tumor cells. Thus, nanosized curcumin has become extremely attractive in immune combinatorial therapies for esophageal adenocarcinoma. Interestingly, normal esophageal HET-1A cells have shown resistance to nanocurcumin, indicating the safety profile of this herbal nanoparticle to the normal cells [47].

Likewise, the differential cytotoxicity of the nanocurcumin was evident in the current study, where normal gingival fibroblasts revealed poor sensitivity to nanocurcumin. This concept has been invested in using nanocurcumin as a natural chemo-preventive candidate against the development of breast cancer in a mammary carcinogenesis model. The rats co-treated with nanocurcumin during cancer induction have shown resistance to developing cancer with pronounced apoptotic tissue reaction [48].

For differentiation between cell death mechanisms, nanocurcumin proved its cytotoxicity to be apoptotic cell death using Annexin V-FITC/PI stain. The results were sequential, where nanocurcumin revealed early apoptosis after four h, which shifted to late apoptosis after 24 h. On the other hand, DOX showed its early and late cytotoxic effects as necrosis. Likewise, previous reports have shown the early and late apoptotic effect of nanocurcumin combating a wide array of cancer cell lines, including breast cancers MDA-MB-231 [50], primary glioblastoma U87-MG [51], laryngeal cancer Hep-2 [52]. Curcumin group results were of conflict image attributed to the organic solvent, where rude herbal candidate induced late apoptosis mixed with necrosis after four h. Meanwhile, most oral cancer cells were necrotic after 24 h curcumin treatment. Using the AO/EB dual staining technique, curcumin has revealed similar late apoptotic and necrotic effects against lung adenocarcinoma cell line A549 [14]. However, in combating MDA-MB-231 and MCF7, curcumin has demonstrated an apoptotic effect. After 48 h, the pro-apoptotic Bax protein has overexpressed with under expression of the anti-apoptotic Bcl-2 [53].

The bioavailability of the potent apoptotic nanocurcumin showed further proof of its inhibitory impact in diminishing the migratory capacity of SCC4 cells comparable to the DOX. However, the latter chemotherapeutic suppressive effect was necrotic rather than apoptotic. In contrast, curcumin showed minimal inhibitory effect without preserving the patency of the wound gap as efficiently as nanoparticles due to crystals precipitated with acetone, hindering curcumin from exerting anti-migratory effects. In addition to SCC4, dendrosomal nanocurcumin has demonstrated a potent anti-migratory effect comparable to DOX against metastatic breast cancer MDA-MB-231. Furthermore, combining both dendrosomal nanocurcumin with DOX has exhibited a synergistic anti-migratory effect compared with monotherapy [50]. Moreover, curcumin has reported a notable anti-migratory effect when formulated in niosome nanoparticles against the human glioblastoma stem-like cells (GSCs) compared with the bulk form of curcumin [54]. Despite the minimal inhibitory effect of curcumin reported against SCC4 in the current study, hydrazinobenzoyl curcumin has demonstrated a potent dose and time-dependent anti-migratory effect against human glioblastoma cell lines (U87MG and U373MG) [55].

At the cellular homeostasis level, the ability of our curcumin nanoplatform in oxidative stress induction tilted towards trapping the released ROS, proving the antioxidant power of the bioavailable soluble nanocurcumin. However, the native curcumin particles showed more affinity towards MDA-mediated lipid peroxidation. In other human hepatoma cell lines, nanocurcumin has scavenged the free radicles more efficiently than the curcumin complement [56]. Further in vivo models of global cerebral ischemia and Parkinson’s disease have displayed increased antioxidant activity with the anti-lipid peroxidation improvement by the different nanocurcumin platforms [57, 58]. Moreover, the bioavailable nanocurcumin has enhanced antioxidant activities, detoxifying circulatory carcinogenic dimethylhydrazine [59]. Additionally, curcumin nanoparticles acting as an antidote have stabilized the oxidative status against liver mitochondria-induced toxicity [60].

Beyond its influential properties, curcumin possesses self-luminescence, the characteristic optical property that qualifies curcumin as one of the intensively used loading dyes, tagging other therapies to allow their tracing. However, the acetone dissolved the cancer cell membrane leading to a rapid influx of the curcumin particles, giving intense fluorescent signals with delirious cytological alterations. Therefore, the gradual uptake of the counter nanoparticles facilitated their uptake localization with an efficient tracing of the apoptotic effect.

In targeting breast cancer cells by influenza virosomes, the hydrophilic nanocurcumin has tagged the target vehicle by integrating it within the viral membrane. The luminescent nanocurcumin has confirmed the high efficacy of site-specific virosomes-targeting with cytoplasmic localization of the potential drug delivery vehicle [61]. Furthermore, the brightness of curcumin fluorescent has qualified its substitution for the phosphors used for bio hybrid light-emitting diodes. Curcumin offers a metal-free, rare-earth phosphor-free, ecofriendly, and cost-competitive alternative for labeling DNA bio-scaffold for visible light emission [62].

In conclusion, the PVP-stabilized nanocurcumin precipitated by solvent anti-solvent technique is of a monomodal dispersed population of high yielding capacity. Owe to the enhanced bioavailability, the nanocurcumin acquired a comparable anti-tumorigenic effect to chemotherapeutic. The desirable apoptotic cell death of the hydrophilic nano-herb potentiates its cytotoxic efficacy, rather than necrotic cytotoxicity aggravating immune reaction. Moreover, the extraordinary luminescence of nanocurcumin qualifies it to serve as a double theranostic agent. However, for implementing this promising herbal nano-candidate as a substitutional complementary therapy to avoid the delirious side effect of the traditional cancer treatment modalities as well as for proper understanding of the complex molecular pathways of the different oral cancers sub-phenotypes, further in vitro studies on wide spectra of oral cancer cell lines are needed. Additionally, further in vivo studies of different carcinogenesis models are highly recommended as nanocurcumin biodistribution and pharmacokinetics are important determinants for the translational of this nano-herb to the clinical side.

## Supplementary Information

**Additional file 1.** 

## Data Availability

All data generated or analyzed during this study are included in this published article.

## Abbreviations

AO/EB: Acridine orange/ethidium bromide co staining
DMEM: Dulbecco's Modified Eagle's Medium
DMSO: Dimethyl sulfoxide
DOX: Doxorubicin
FITC: Fluorescein isothiocyanate
FTIR: Fourier transforms infrared spectrometer
FBS: Fetal bovine serum
GSH: Reduced glutathione
H_2_O_2_: Hydrogen peroxide
IC_50_: Half-maximal inhibitory concentration
MCF7: Metastatic breast adenocarcinoma cell line
MDA: Malondialdehyde
MDA-MB-231: Triple negative breast adenocarcinoma cell line
MTT: 3-(4,5-Dimethythiazol-2-yl)-2,5-diphenyltetrazolium bromide
NO: Nitric oxide
PBS: Phosphate-buffered saline
PDI: Polydispersity index
PI: Propidium iodide
PVP: Polyvinylpyrrolidone
ROS: Reactive oxygen species
SCC4: Oral squamous cell carcinoma cell line
TAC: Total antioxidant capacity
TEM: Transmission electron microscope
