# Supplementary material for: The theranostic potentialities of bioavailable nanocurcumin in oral cancer management
Source: BMC Complement Med Ther. 2022 Nov 24;22:309. doi: 10.1186/s12906-022-03770-3 (PMC9685877; doi:10.1186/s12906-022-03770-3)
Supplement: Supplementary file 1 — Additional file 1. [file 12906_2022_3770_MOESM1_ESM.pdf]

The theranostic potentialities of bioavailable nanocurcumin in oral cancer management

Supplementary material

Materials and Methods

2.2. Synthesis and characterization of curcumin nanoparticles

For assessing water-solubility of the curcumin nanoparticles versus curcumin powder, 10 mg of freeze-dried nanocurcumin powder was dissolved in 1 ml DIH_2O, with comparing the solubility of an equivalent dose of curcumin powder. Furthermore, the nanocurcumin solubility in Dulbecco's Modified Eagle's Medium (DMEM) was assessed, as DMEM is the culture medium used to cultivate oral cancer cell line in the second phase of the current study.

To reach the extinction coefficient (ε) of the stabilized curcumin nanoparticles, the standard curve was performed, by preparing serial concentrations of (5, 4, 3, 2, and 1 mg/ml) from a stock solution of 15 mg lyophilized nanocurcumin dissolved in 3 mL DIH_2O.

For plotting the standard curve of the curcumin nanoparticles, UV–visible spectrophotometer was done on a nanodrop to obtain the absorption of each dilution. Then the absorbance was plotted on the y-axis and concentration on the x-axis to gain the equation of Beer-Lambert law ($c = \lambda / \varepsilon$), where c is the concentration in molarity, λ is the absorption of each dilution, and ε is the extinction coefficient.

Results

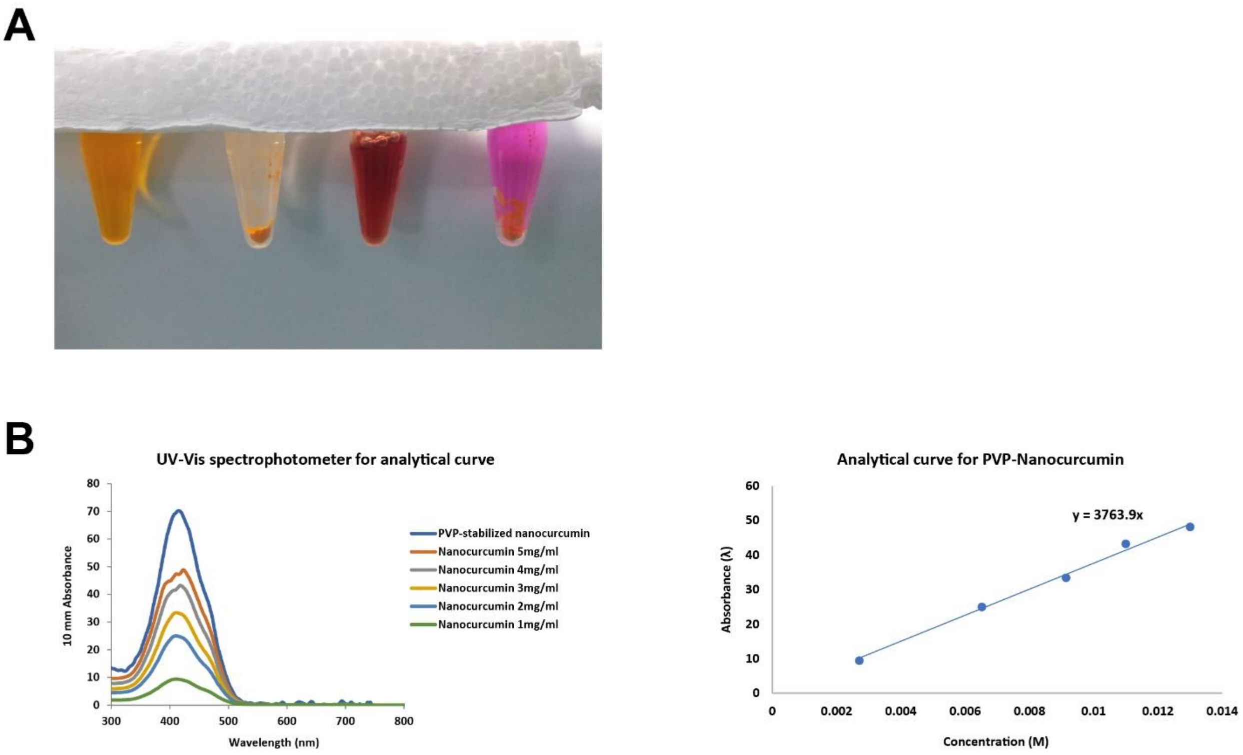

Supplementary figure 1. Physical properties and the analytical curve of 0.5% PVP-stabilized nanocurcumin particles. (A) The solubility test reveals the high solubility of nanocurcumin versus curcumin in DIH_2O and DMEM, which results in a highly concentrated nanosuspension of ~36.7 mM calculated from the analytical curve in (B).

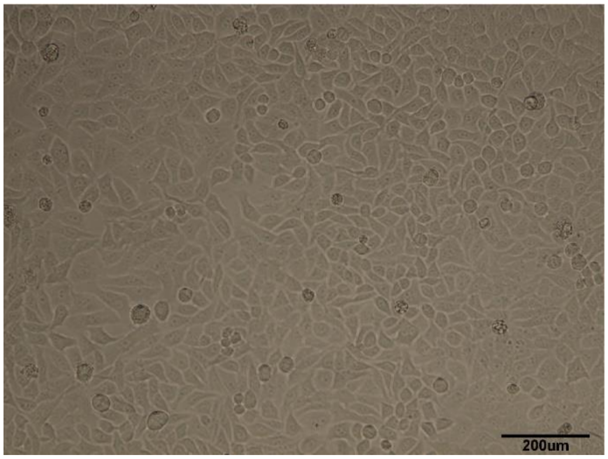

Supplementary figure 2. An inverted light microscope photomicrograph of the sham group. The untreated prickle squamoid epithelial cancer cells –retrieved from 55 years old male patient with tongue squamous cell carcinoma- showed large centrally located vesicular nuclei and prominent nucleoli.

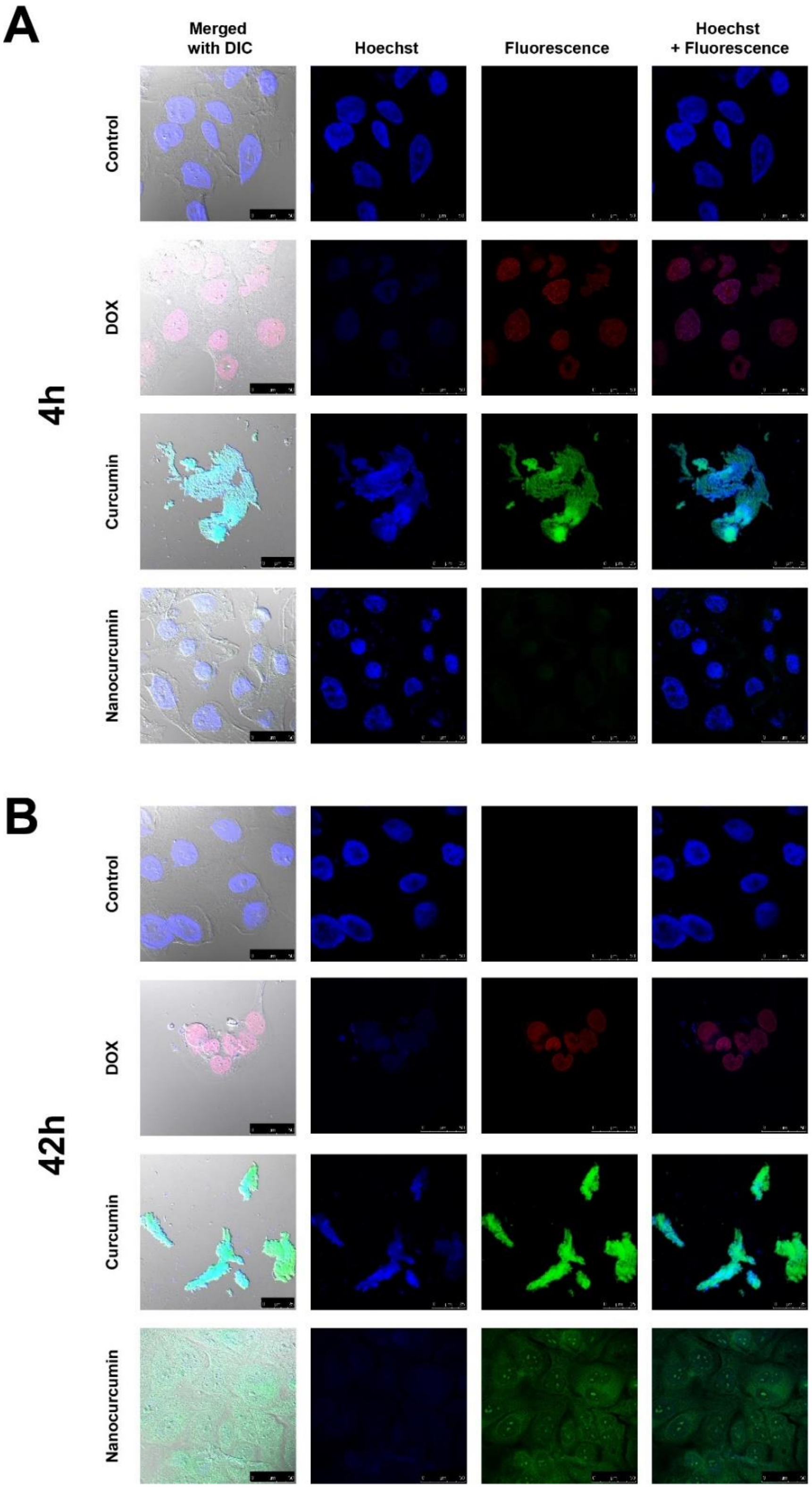

Supplementary figure 3. The collected channels of the representative confocal microscopy images of both time intervals 4 and 24 h incubation with different treatment modalities.
